# Robotic‐assisted total knee arthroplasty achieves superior early postoperative outcomes compared to conventional techniques in patients with severe varus

**DOI:** 10.1002/jeo2.70468

**Published:** 2025-10-28

**Authors:** Shuaijing Feng, Pengfei Xing, Junsong Qu, Jiarong Guo, Jie Song, Haoran Li, Huaiyu Jiang, Huanting Pi, Tao Huang

**Affiliations:** ^1^ Department of Orthopedics The First Hospital of China Medical University Shenyang City Liaoning Province China

**Keywords:** CTKA, knee varus, patient‐reported outcome measures, RATKA, soft tissue release

## Abstract

**Purpose:**

This study aimed to compare the clinical efficacy of robot‐assisted total knee arthroplasty (RATKA) and conventional total knee arthroplasty (CTKA) in patients with varying degrees of varus, and to determine the indications and advantages of RATKA.

**Methods:**

A retrospective analysis was conducted on clinical data from patients with knee osteoarthritis who underwent either RATKA or CTKA. Univariate and multivariate linear regression analyses were performed to identify factors influencing postoperative hip–knee–ankle (HKA) angle deviation. Based on the degree of preoperative HKA angle deviation, patients were grouped into mild varus (HKA angle deviation <10°) and severe varus groups (HKA angle deviation ≥10°). Postoperative outcomes of the two surgical techniques were compared in patients with varying degrees of varus. These included lower limb alignment parameters such as HKA, posterior slope angle (PSA), medial proximal tibial angle (MPTA), and femoral distal lateral angle (FDLA); clinical functional scores including Hospital for Special Surgery score (HSS), Western Ontario and McMaster Universities Osteoarthritis Index (WOMAC), visual analog scale for pain (VAS), and range of motion (ROM); as well as operative time and the proportion of cases requiring soft tissue release. Alignment parameters and functional scores were assessed 1 week before surgery and 3 months postoperatively.

**Results:**

A total of 218 patients were included. The mean ages of the RATKA and CTKA groups were 64.9 ± 6.5 years and 65.1 ± 5.7 years, respectively. The RATKA group included 15 males and 69 females, while the CTKA group included 29 males and 105 females. Multivariate analysis identified preoperative HKA angle deviation as the most important predictor of postoperative HKA alignment, with each 1° increase in preoperative deviation associated with an approximate 0.150° increase postoperatively. In patients with mild varus, there were no significant differences in alignment restoration or clinical scores between RATKA and CTKA. However, in the severe varus groups, RATKA demonstrated significantly better outcomes in terms of HKA, PSA, MPTA and FDLA alignment parameters, lower radiographic outlier rates, reduced need for soft tissue release, and superior HSS, WOMAC and VAS scores (*p* < 0.05).

**Conclusion:**

RATKA provides greater clinical benefits in patients with severe varus, offering more accurate limb alignment, improved implant positioning, and better short‐term functional outcomes. RATKA should be preferentially considered for patients with substantial preoperative HKA angle deviation.

**Level of Evidence:**

Level Ⅲ, retrospective cohort study.

**Abbreviations**
CTKA: conventional total knee arthroplasty
FDLA: femoral distal lateral angle
HKA: hip–knee–ankle
HSS: hospital for special surgery
MPTA: medial proximal tibial angle
PSA: posterior slope angle
RATKA: robotic‐assisted total knee arthroplasty
ROM: range of motion
SEA: surgical epicondylar axis
VAS: visual analogue scale
WOMAC: Western Ontario and McMaster universities osteoarthritis index

## INTRODUCTION

Conventional total knee arthroplasty (CTKA) has been proven effective in relieving pain, correcting deformities and restoring mobility in patients with knee osteoarthritis [7, 11, 19]. However, CTKA still faces considerable challenges when treating patients with severe varus, particularly in terms of lower limb alignment, prosthesis positioning and soft tissue balancing—factors that directly affect the long‐term surgical outcomes and the quality of patient recovery [12, 27]. Although CTKA is widely used, follow‐up studies have reported postoperative patient satisfaction rates ranging from 82% to 89% [3, 6, 9]. Furthermore, data from global joint registries indicate revision rates of 8.91% at 5 years and 12.9% at 10 years following CTKA [15], highlighting the considerable potential for improvement in this procedure.

Compared with CTKA, robot‐assisted total knee arthroplasty (RATKA) utilizes precise navigation and three‐dimensional reconstruction technologies to achieve more accurate prosthesis positioning, improved limb alignment and reduced need for soft tissue release [8, 31], RATKA also enhances bone‐cutting accuracy, minimizes the risk of femoral intramedullary canal injury and increases the overall predictability and safety of the procedure [10, 14, 15, 23, 30]. Nonetheless, the existing evidence on this technology remains controversial [32], with some studies suggesting that robotic systems do not offer a significant advantage over traditional CTKA in certain scenarios [13, 35].

Although research on RATKA has been increasing, most studies remain focused on direct comparisons with CTKA, with limited consideration of individual patient characteristics prior to surgery [10, 14, 23, 30]. This study aimed to evaluate the clinical effectiveness of RATKA in patients with varying degrees of knee varus. We hypothesize that RATKA and CTKA may differ in their effectiveness regarding mechanical alignment restoration, implant positioning accuracy, soft tissue preservation and early postoperative functional improvement in patients with varying degrees of varus. Furthermore, these differences may manifest as distinct advantages depending on the severity of the varus.

## METHODS

### Study design

This retrospective clinical study included patients who underwent TKA between January 2021 and January 2025. The inclusion criteria were as follows: (1) End‐stage knee osteoarthritis, rheumatoid arthritis or traumatic arthritis that has failed conservative treatment for 6 months to 1 year, or has reached Kellgren–Lawrence grade 3. (2) Patients aged 50 years or older. (3) Good local skin condition around the knee, without any signs of infection. The exclusion criteria were as follows: (1) Patients with serious comorbidities, including metabolic bone diseases, neurological dysfunction, poorly controlled diabetes, or severe cardiopulmonary conditions such as COPD (GOLD stage III–IV, FEV₁ < 50%) or heart failure (ACC/AHA stage D, NYHA class IV). (2) Previous similar surgeries performed on the same knee. (3) High risk of infection. (4) Diseases affecting lower limb nerve function, such as Parkinson's disease and severe lumbar disc herniation. (5) Patients with valgus knee.

In this study, univariate and multivariate linear regression analyses were first performed to identify key factors influencing postoperative hip–knee–ankle (HKA) angle deviation. Based on the results, patients were grouped according to their preoperative HKA angle deviation and surgical technique, and clinical outcomes of RATKA and CTKA were further compared across different severities of varus. Primary outcome measures included restoration of the mechanical axis of the lower limb, prosthesis alignment accuracy, and postoperative functional scores. Additionally, the number and anatomical sites of intraoperative soft tissue releases were analyzed to further assess the effectiveness of RATKA in achieving soft tissue balance. This approach allows for an evaluation of the clinical applicability of RATKA in patients with varying degrees of varus and helps identify the groups most likely to benefit from this technology.

A total of 267 cases were initially collected for this study, and after applying inclusion and exclusion criteria, 218 patients were ultimately included. Among them, 84 patients were enroled in the RATKA group and 134 patients were enroled in the CTKA group. The patients were further divided into four groups based on the preoperative HKA angle deviation: group A (RATKA, HKA angle deviation <10°, mild varus, 43 patients), group B (RATKA, HKA angle deviation ≥10°, severe varus, 41 patients), group C (CTKA, HKA angle deviation <10°, mild varus, 68 patients), and group D (CTKA, HKA angle deviation ≥10°, severe varus, 66 patients). An HKA angle <180° indicates knee varus, while >180° indicates knee valgus. This study grouped patients based on the degree of knee varus. Currently, HKA angle deviation <10.0° is generally classified as mild varus, while HKA angle deviation ≥10.0° is considered indicative of severe varus alignment [22]. The study protocol was approved by the Ethics Committee of the First Hospital of China Medical University (Approval No. [2024]1075).

**Figure 1:**
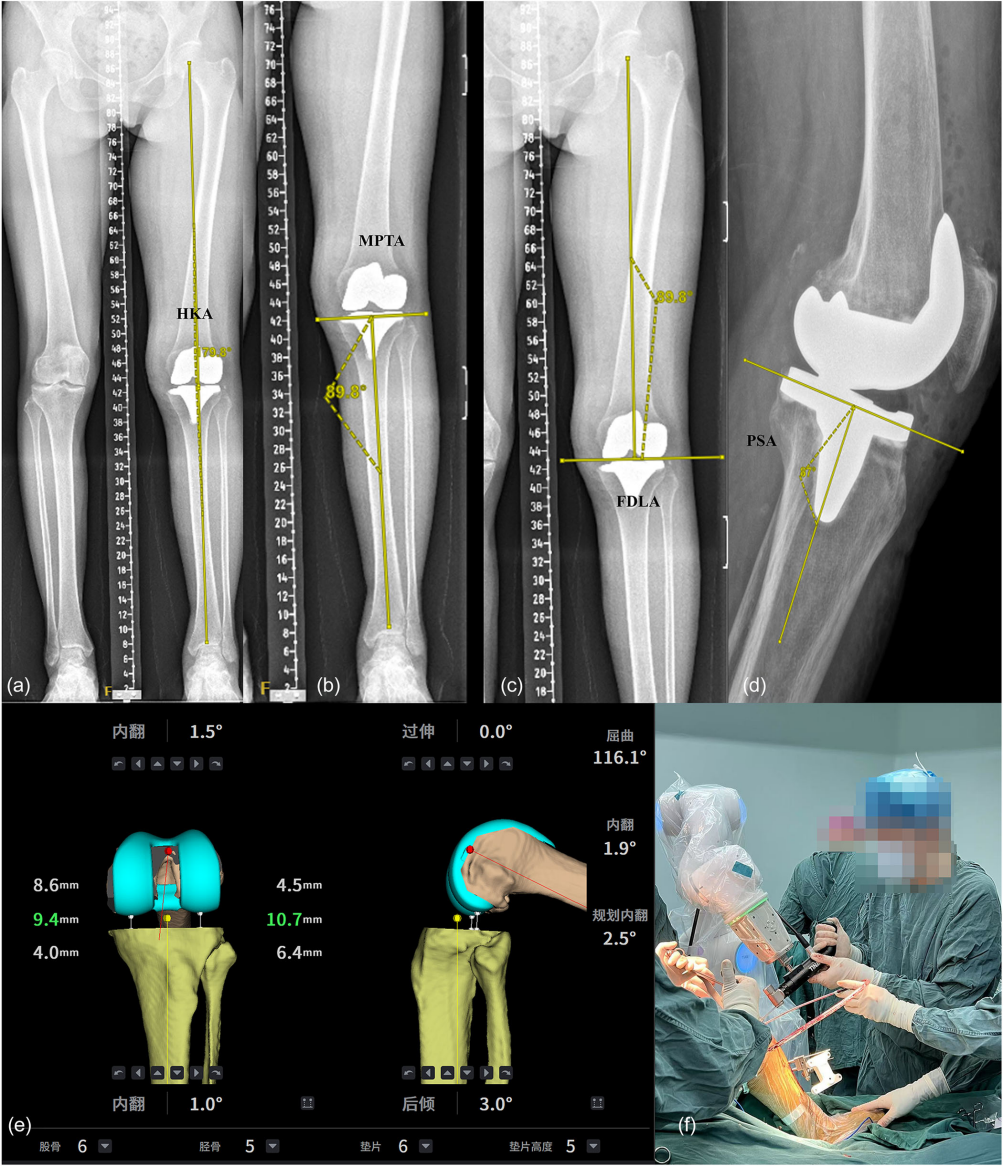
Prosthesis position measurement on DR films after RATKA and intraoperative diagram of TINAVI robotic system. (a) HKA; (b) MPTA; (c) FDLA; (d) PSA; (e) Intraoperative screenshot of the TINAVI system; (f) TINAVI robot‐assisted osteotomy. FDLA, femoral distal lateral angle; HKA, hip–knee–ankle; MPTA, Medial proximal tibial angle; PSA, posterior slope angle; RATKA, robotic‐assisted total knee arthroplasty.

Baseline characteristics, including age, sex, body weight and body mass index (BMI), were collected for all enroled patients. Preoperative and postoperative radiographs of the entire lower limbs in anterior‐posterior (AP) view and knee joints in both AP and lateral views were taken. As shown in Figure 1, imaging measurement indicators included: (1) HKA angle: Angle between the mechanical axes of the femur and tibia (normal: 180° ± 3°, Figure 1a), HKA angle deviation was defined as 180° minus the measured HKA angle. (2) Medial proximal tibial angle (MPTA): Angle between the tibial mechanical axis and proximal tibial joint line (normal: 90° ± 3°, Figure 1b). (3) Femoral distal lateral angle (FDLA): Angle between the femoral mechanical axis and distal femoral joint line (normal: 90° ± 3°, Figure 1c). (4) Posterior slope angle (PSA): Angle between the horizontal plane and the tibial plateau's posterior slope (ideal: 3°); [20] contributes to joint stability (Figure 1d).

Additionally, the hospital for special surgery (HSS) score, Western Ontario and McMaster Universities Osteoarthritis Index (WOMAC) score, visual analogue scale (VAS) score, and range of motion (ROM) were assessed 1 week before surgery and 3 months after surgery. Intraoperative soft tissue release data were also collected. The assessment of intraoperative soft tissue release was primarily based on joint gap balance and knee stability, combined with the use of gap measurement spacers, trial prostheses and soft tissue tension testing to determine whether release was necessary. Specific criteria for performing soft tissue release included limited joint mobility, uneven joint gaps or inadequate intraoperative knee stability.

### Surgical procedures

The TINAVI robotic system (TINAVI Medical Technologies Co., Ltd.) is a high‐precision surgical assistant composed of a robotic arm, intraoperative navigation system and touchscreen control interface. It constructs a patient‐specific 3D bone model based on preoperative CT scans to formulate a surgical plan for osteotomy and prosthesis placement. Intraoperatively, the system integrates infra‐red tracking to monitor the real‐time positions of bones and instruments. The robotic arm executes bone cuts along predefined paths with built‐in constraints to prevent deviation. Additionally, the system continuously records flexion–extension gaps and soft tissue tension to assist in intraoperative adjustments and prosthesis alignment. The procedure consisted of four main steps: (1) Preoperative planning and 3D modelling: All patients underwent high‐resolution CT scans of the lower extremity (including the hip, knee and ankle). The imaging data were imported into the robotic system software to construct a patient‐specific 3D anatomical model. Based on this model, an individualized surgical plan was generated, including implant sizing and positioning for both femur and tibia, bone resection depth and angles, personalized lower limb alignment strategies, as well as flexion‐extension gap balancing and soft tissue tension assessments. (2) Intraoperative preparation and anatomical registration: Following exposure of the knee joint, optical trackers were fixed to the femur and tibia. The surgeon performed registration of anatomical landmarks according to navigation prompts, enabling accurate matching between the virtual model and the actual anatomy. The system automatically computed and displayed real‐time parameters such as limb alignment, range of motion and gap measurements. (3) Bone resection and soft tissue balancing: Under robotic navigation, the femur and tibia were resected with a robotic arm‐controlled saw, precisely following the preoperative plan. To prevent injury to critical anatomical structures, the system included a safety mechanism that automatically halted saw movement upon approaching predefined high‐risk zones. The system provided real‐time visualization of limb alignment, joint space, implant orientation and soft tissue balance in both flexion and extension positions. Adjustments to implant positioning could be made intraoperatively to optimize mechanical alignment. Furthermore, the system simulated and quantified the biomechanical effects of various implant positions on joint stability and soft tissue tension to guide intraoperative decision‐making. Minimal soft tissue release was performed only when necessary. (4) Trial implantation, final implantation, and wound closure: After bone cuts were completed, trial components were inserted to assess and quantify knee kinematics, dynamic alignment, implant stability, and patellar tracking. The intraoperative diagram of TINAVI robot is shown in Figure 1e,f.

Both the RATKA and CTKA groups underwent surgery via a standard medial parapatellar approach. In the CTKA group, during distal femoral resection, the average valgus angle was set to 5–7°. An average external rotation angle of 3° was set during posterior condyle resection. However, for patients with severe posterior condyle wear, the resection angle was determined by combining the surgical epicondylar axis (SEA) and the anteroposterior axis.

All patients in the four groups underwent surgery with a lower limb tourniquet inflated to 280 mmHg. Thirty minutes before surgery, all patients received standard prophylactic measures: 1.0 g cefuroxime and 1.0 g tranexamic acid. All surgeries utilized posterior stabilized knee prosthesis (Johnson & Johnson). Postoperatively, all patients received routine infiltration of the incision site with lidocaine to alleviate postoperative pain and facilitate rehabilitation. This standardized perioperative management protocol ensured consistent care across all groups, contributing to optimal surgical outcomes and patient recovery. All surgeries in this study were performed by a single orthopaedic surgeon with over 10 years of experience in TKA and an annual case volume of more than 100 procedures.

### Statistical analysis

To evaluate the normal distribution of continuous variables, the Shapiro–Wilk test was employed. Normally distributed data were expressed as mean ± SD, while skewed data were presented as median (interquartile range). The significance of normally distributed data was assessed using the Student's *t*‐test, whereas the Mann–Whitney *U* test was used for skewed data. The chi‐square test was applied to assess the significance of categorical variables. Postoperative HKA angle deviation was used as the dependent variable. A significance level (*α*) of 0.05 was set for statistical testing. Univariate linear regression analyses were first performed using the patients' overall demographic characteristics and radiographic parameters. Variables with a *p* < 0.05 in the univariate analyses were subsequently included in a multivariate linear regression model to identify independent predictors of postoperative HKA angle deviation. All data were analyzed using SPSS statistical software, version 25.0 (SPSS). The significance level was defined as *p* < 0.05.

## RESULTS

### Preoperative patient data analysis

The baseline characteristics of the included patients were well balanced, with no significant differences between groups in key indicators such as age, sex, BMI, and preoperative HKA angle. The results are summarized in Table 1.

**Table 1:** Baseline characteristics of the included cohort.

Variable	RATKA	CTKA	*p* value
Number of patients	84	134	‐
Age (years)	64.9 ± 6.5	65.1 ± 5.7	ns
Gender (case, men/women)	15/69	29/105	ns
BMI (kg/m^2^)	26.4 ± 2.9	27.1 ± 3.0	ns
Operational side (left/right)	39/45	62/72	ns
HKA angle deviation (°)	9.5 ± 2.2	9.8 ± 2.8	ns
HSS	57.3 ± 5.5	56.2 ± 5.0	ns
WOMAC	58.9 ± 7.2	58.5 ± 7.0	ns
VAS	3 (3–4)	4 (3–4)	ns
ROM (°)	91.3 ± 6.5	89.9 ± 8.7	ns

Abbreviations: BMI, body mass index; CTKA, conventional total knee arthroplasty; HKA, hip–knee–ankle; HSS, hospital for special surgery; ns, not significant; RATKA, robot‐assisted total knee arthroplasty; ROM, range of motion; VAS, visual analogue scale; WOMAC, Western Ontario and McMaster Universities Osteoarthritis Index.

### Factors influencing postoperative HKA angle deviation

To identify factors associated with postoperative HKA angle deviation, we first performed univariate linear regression analyses using demographic variables (sex, age, BMI, operational side and surgical technique), radiographic parameters (preoperative HKA angle deviation), and clinical scores (HSS, WOMAC, VAS and ROM). The results indicated that surgical technique, preoperative HKA angle deviation, preoperative HSS score, preoperative WOMAC score, and preoperative VAS score were potential key contributors to postoperative HKA angle deviation (Table 2).

**Table 2:** Results of univariate linear regression analysis.

Variables	*B*	*β*	95% CI	*t*	*p* value
Age (years)	0.016	0.078	−0.01 to 0.042	1.152	ns
Gender	0.249	0.083	−0.150 to 0.648	1.229	ns
BMI (kg/m^2^)	−0.008	−0.020	−0.063 to 0.047	−0.297	ns
Operational side (left/right)	0.083	0.035	−0.239 to 0.405	0.612	ns
Surgical technique	0.496	0.201	0.172 to 0.819	3.021	[**]
HKA angle deviation (°)	0.177	0.379	−0.119 to 0.235	6.018	[**]
HSS	−0.054	−0.235	−0.085 to −0.024	−3.550	[**]
WOMAC	0.023	0.133	0.000 to 0.045	1.975	[**]
VAS	0.296	0.190	0.091 to 0.502	2.845	[*]
ROM (°)	−0.020	−0.130	−0.040 to 0.001	−1.921	ns

Abbreviations: BMI, body mass index; CO, confidence interval; HKA, hip–knee–ankle; HSS, hospital for special surgery; ns, not significant; ROM, range of motion; VAS, visual analogue scale; WOMAC, Western Ontario and McMaster Universities Osteoarthritis Index.

* *p* < 0.05

** *p* < 0.01.

Surgical technique, preoperative HKA angle deviation, preoperative HSS score, preoperative WOMAC score, and preoperative VAS score were included in a multivariate linear regression model. The analysis revealed that both surgical technique and preoperative HKA angle deviation were statistically significant predictors of postoperative HKA angle deviation (Table 3). According to the regression model, each 1° increase in preoperative HKA angle deviation was associated with a 0.150° increase in postoperative deviation. Additionally, switching from RATKA to CTKA was associated with a 0.410° increase in postoperative HKA angle deviation. Other variables did not reach statistical significance.

**Table 3:** Results of multivariate linear regression analysis.

Variables	*B*	*β*	95% CI	*t*	*p* value
Surgical technique	0.410	0.166	0.111 to 0.708	2.706	[**]
HKA angle deviation (°)	0.150	0.321	0.091 to 0.209	5.027	[**]
HSS	−0.28	−0.123	−0.057 to 0.000	−1.954	ns
WOMAC	0.019	0.114	−0.001 to 0.040	10.862	ns
VAS	0.130	0.083	−0.063 to 0.323	1.328	ns

Abbreviations: CI, confidence interval; HKA, hip–knee–ankle; HSS, hospital for special surgery; ns, not significant; VAS, visual analogue scale; WOMAC, Western Ontario and McMaster Universities Osteoarthritis Index.

** *p* < 0.01.

Given that preoperative HKA angle deviation and surgical technique were identified as the most influential factor, patients were subsequently grouped into four groups based on the surgical technique and severity of varus: group A (RATKA, HKA angle deviation <10°, mild varus), group B (RATKA, HKA angle deviation ≥10°, severe varus), group C (CTKA, HKA angle deviation <10°, mild varus), and group D (CTKA, HKA angle deviation ≥10°, severe varus) [22], for further comparative analysis of surgical outcomes.

### Comparison of postoperative baseline data and scoring between patients with mild and severe varus

#### Analysis of postoperative baseline data and scoring in the mild varus groups

**Figure 2:**
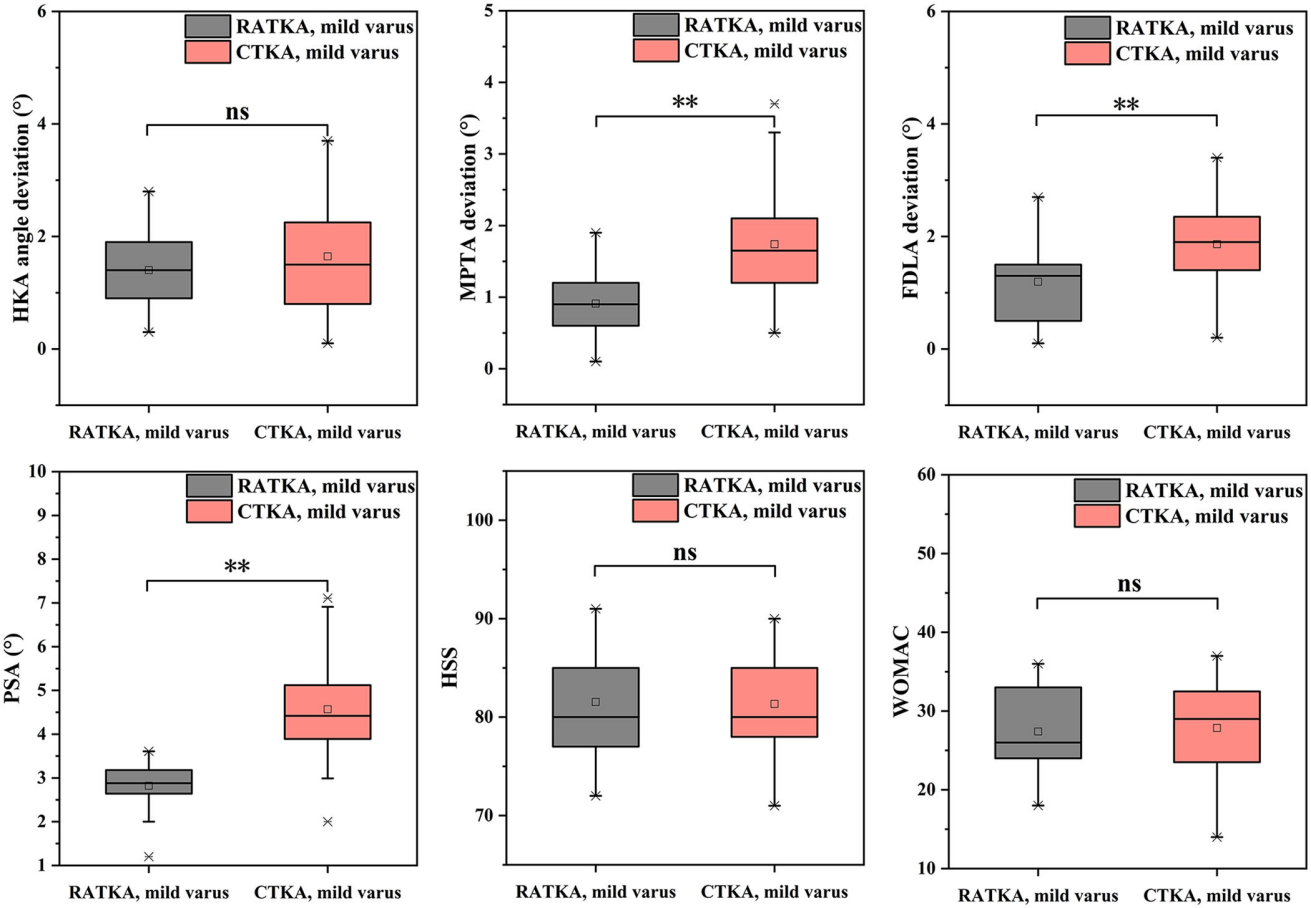
Key postoperative information for mild varus groups. ∗*p* < 0.05; **p < 0.01. CTKA, conventional total knee arthroplasty; ns, not significant; RATKA, robot‐assisted total knee arthroplasty.

In the mild varus groups, the postoperative HKA angle deviation was 1.4 ± 0.7° for the RATKA group and 1.6 ± 1.0° for the CTKA group, with no statistical difference (*p* > 0.05). Postoperative PSA values were 2.8 ± 0.5° for RATKA and 4.6 ± 1.1° for CTKA, showing a highly significant difference (*p* < 0.01). The postoperative MPTA deviation was 0.9 ± 0.4° for RATKA and 1.7 ± 0.7° for CTKA, also demonstrating a significant difference (*p* < 0.01). Postoperative FDLA deviation was 1.2 ± 0.7° for RATKA and 1.9 ± 0.6° for CTKA, with a statistically significant difference (*p* < 0.01). The average surgical time for the RATKA and CTKA groups were 115.6 ± 6.3 minutes and 94.1 ± 11.2 min, respectively, with a statistical difference (*p* < 0.01). The longer surgical time in the RATKA group is consistent with previous studies [24, 25]. Three months postoperatively, follow‐up assessments revealed no statistical differences between the two groups in HSS scores, WOMAC scores, VAS scores, and ROM (*p* > 0.05). Table 4 summarized the postoperative data for both groups, and the key postoperative information for both groups are also shown in Figure 2.

**Table 4:** Postoperative outcome analysis in the mild varus group.

Variable	RATKA (mild)	CTKA (mild)	*p* value
HKA angle deviation (°)	1.4 ± 0.7	1.6 ± 1.0	ns
PSA (°)	2.8 ± 0.5	4.6 ± 1.1	[**]
MPTA deviation (°)	0.9 ± 0.4	1.7 ± 0.7	[**]
FDLA deviation (°)	1.2 ± 0.7	1.9 ± 0.6	[**]
Time (min)	115.6 ± 6.3	94.1 ± 11.2	[**]
HSS	81.5 ± 5.3	81.3 ± 5.1	ns
WOMAC	27.4 ± 5.2	27.9 ± 5.9	ns
VAS	1 (1–1)	1 (1–2)	ns
ROM (°)	106.9 ± 4.4	106.1 ± 4.8	ns

Abbreviations: CTKA, conventional total knee arthroplasty; FDLA, femoral distal lateral angle; HKA, hip–knee–ankle; HSS, hospital for special surgery; MPTA, medial proximal tibial angle; ns, not significant; PSA, posterior slope angle; robot‐assisted total knee arthroplasty; VAS, visual analogue scale; WOMAC, Western Ontario and McMaster Universities Osteoarthritis Index.

** *p* < 0.01.

#### Analysis of postoperative baseline data and scoring in the severe varus groups

**Figure 3:**
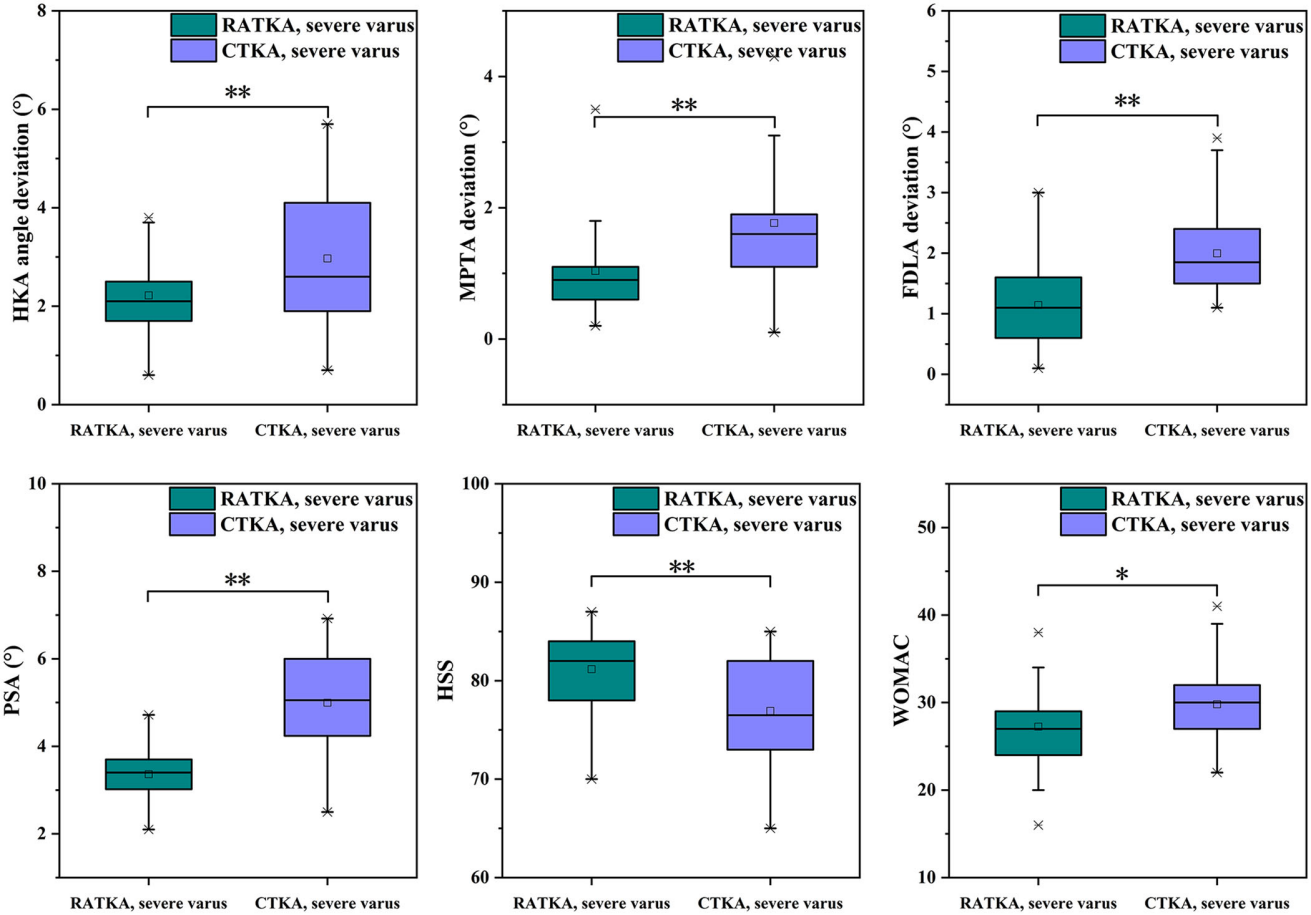
Key postoperative information for severe varus groups. ∗*p* < 0.05; **p < 0.01. CTKA, conventional total knee arthroplasty; MPTA, medial proximal tibial angle; RATKA, robot‐assisted total knee arthroplasty.

For the severe varus groups, the postoperative HKA angle deviation in the RATKA and CTKA groups was 2.2 ± 0.7° and 3.0 ± 1.4°, respectively, with a statistical difference (*p* < 0.01). Postoperative PSA values were 3.4 ± 0.6° and 5.0 ± 1.0°, respectively, showing a highly significant difference (*p* < 0.01). The postoperative MPTA deviation in the RATKA group was 1.0 ± 0.7, compared to 1.8 ± 0.9° in the CTKA group, also demonstrating a significant difference (*p* < 0.01). The postoperative FDLA deviation in the RATKA group was 1.1 ± 0.7°, whereas the CTKA group had 2.0 ± 0.6°, with a statistically significant difference (*p* < 0.01). The average surgical time for the RATKA and CTKA groups were 119.1 ± 11.5 min and 102.1 ± 10.0 min, respectively, with a statistical difference (*p* < 0.01). Compared to the CTKA group, the RATKA group had a longer surgical time. Unlike the mild varus group, the RATKA group showed better performance in HSS scores, WOMAC scores, VAS scores, and ROM, at 3 months postoperatively in severe varus patients. Table 5 summarized the postoperative data for both groups, and the key postoperative information for both groups are also shown in Figure 3.

**Table 5:** Postoperative outcome analysis in the severe varus group.

Variable	RATKA (severe)	CTKA (severe)	*p* value
HKA angle deviation (°)	2.2 ± 0.7	3.0 ± 1.4	[**]
PSA (°)	3.4 ± 0.6	5.0 ± 1.0	[**]
MPTA deviation (°)	1.0 ± 0.7	1.8 ± 0.9	[**]
FDLA deviation (°)	1.1 ± 0.7	2.0 ± 0.6	[**]
Time (min)	119.1 ± 11.5	102.1 ± 10.0	[**]
HSS	81.2 ± 4.1	76.9 ± 5.2	[**]
WOMAC	27.2 ± 4.9	29.8 ± 4.6	[*]
VAS	1 (1–2)	2 (2–2.75)	[*]
ROM (°)	105.6 ± 5.2	100.2 ± 5.4	[**]

Abbreviations: CTKA, conventional total knee arthroplasty; FDLA, femoral distal lateral angle; HKA, hip–knee–ankle; HSS, hospital for special surgery; MPTA, medial proximal tibial angle; PSA, posterior slope angle; RATKA, robot‐assisted total knee arthroplasty; VAS, visual analogue scale; WOMAC, Western Ontario and McMaster Universities Osteoarthritis Index.

* *p* < 0.05

** *p* < 0.01.

### Comparison of radiographic outlier rates between patients with mild and severe varus

As shown in Table 6, the proportion of patients with a postoperative HKA angle deviation beyond 3° was 0% in the RATKA mild varus group and 13.2% in the CTKA mild varus group. In the RATKA severe varus group and CTKA severe varus group, the proportions were 19.5% and 30.3%, respectively. For the MPTA deviation, 0% of patients in the RATKA mild varus group and 8.8% in the CTKA mild varus group exhibited abnormal results (MPTA deviation >3.0°). In the RATKA severe varus group and CTKA severe varus group, 7.3% and 13.6% of patients in both groups demonstrated abnormal results. For the FDLA deviation, patients in the RATKA mild varus group, CTKA mild varus group, and RATKA severe varus group all showed no abnormal values (FDLA deviation >3.0°), whereas 7.6% of patients in the CTKA severe varus group showed abnormal results. Overall, the RATKA group outperformed the CTKA group, particularly in severe varus patients.

**Table 6:** Results of radiographic outlier rates in the mild and severe varus groups.

Group	HKA angle deviation >3°	MPTA deviation >3°	FDLA deviation >3°
A (RATKA, mild varus)	0%	0%	0%
B (RATKA, severe varus)	19.5%	7.3%	0%
C (CTKA, mild varus)	13.2%	8.8%	0%
D (CTKA, severe varus)	30.3%	13.6%	7.6%

Abbreviations: CTKA, conventional total knee arthroplasty; FDLA, femoral distal lateral angle; HKA, hip–knee–ankle; MPTA, medial proximal tibial angle; RATKA, robot‐assisted total knee arthroplasty.

### Comparison of soft tissue release between patients with mild and severe varus

In the mild varus group, the percentage of patients requiring soft tissue release in the RATKA and CTKA groups was 58.1% and 77.9%, respectively (*p* < 0.05). In the severe varus group, the percentage of patients requiring soft tissue release in the RATKA and CTKA groups was 75.6% and 95.5%, respectively, with a statistically significant difference (*p* < 0.01). The comparison data indicate that the number of release cases in the RATKA group was relatively lower. Notably, for patients with severe varus, RATKA demonstrated a greater advantage over CTKA (as shown in Table 7).

**Table 7:** Soft‐tissue release required for equal gap‐balancing.

Technique	RATKA (mild)	CTKA (mild)	*p* value	RATKA (severe)	CTKA (severe)	*p* value
Number of patients	43	68	‐	41	66	‐
Required soft‐tissue Balancing, number	58.1%	77.9%	ns	75.6%	95.5%	[**]
Quadriceps snip	0.0%	0.0%	‐	0.0%	3.0%	ns
Proximal medial release	44.2%	73.5%	[**]	46.3%	75.8%	[**]
Distal medial release	30.2%	44.1%	ns	41.5%	71.2%	[**]
Lateral release	2.3%	10.3%	ns	2.4%	7.6%	ns
Patellar release	0.0%	0.0%	‐	0.0%	3.0%	ns

Abbreviations: CTKA, conventional total knee arthroplasty; ns, not significant; RATKA, robot‐assisted total knee arthroplasty.

** *p* < 0.01.

## DISCUSSION

The main findings of this study are: (1) Preoperative HKA angle deviation and surgical technique are significant factors influencing postoperative HKA deviation. (2) Compared to CTKA, RATKA demonstrates greater advantages in patients with severe varus, including more accurate mechanical axis restoration, implant positioning, and improved functional outcomes. (3) RATKA reduces the need for soft tissue release compared to CTKA, particularly in patients with severe varus.

Accurate restoration of the lower limb mechanical axis following TKA is critical for functional recovery, long‐term prosthesis stability, and overall patient satisfaction. Previous studies have demonstrated that postoperative mechanical alignment should ideally return to a neutral mechanical axis, and deviations exceeding 3° are associated with poorer functional outcomes and higher revision rates [2]. In CTKA, intraoperative bone resections heavily rely on the surgeon's experience. This dependence becomes especially problematic in patients with severe varus, where obscured anatomical landmarks, oblique joint lines, and soft tissue contractures make accurate positioning more difficult and often result in suboptimal alignment restoration [17]. In recent years, the revision rate for CTKA has increased [5]. According to the Australian National Joint Replacement Registry, revision TKA now accounts for 8.3% of all knee arthroplasty procedures [1]. To improve intraoperative accuracy and alignment outcomes, RATKA has been gradually introduced into clinical practice. Several studies have reported potential advantages of RATKA in terms of bone resection precision, mechanical axis alignment, soft tissue tension balancing, and flexion–extension gap control [4, 18, 21, 34]. However, concerns remain regarding longer operative times and increased costs, and its overall clinical superiority remains under debate [13, 32, 35].

This study used postoperative HKA angle deviation as the primary outcome to identify contributing factors via linear regression analysis. Univariate analysis showed that surgical technique, preoperative HKA angle deviation, and baseline HSS, WOMAC and VAS scores were all associated with postoperative alignment. Multivariate analysis further revealed that preoperative HKA angle deviation and surgical technique were the most important independent predictor. Given the clinical importance of achieving postoperative mechanical neutral alignment, patients were grouped into mild and severe varus groups according to preoperative HKA angle deviation. In patients with mild varus, although RATKA showed a certain advantage over CTKA in terms of prosthesis positioning accuracy, there was no significant difference in postoperative HKA alignment between the two groups. However, in patients with severe varus, RATKA demonstrated significantly better outcomes in both postoperative HKA alignment and prosthesis positioning accuracy (*p* < 0.05), indicating its greater efficacy in severe varus patients.

Further radiographic analysis of MPTA and FDLA also demonstrated the benefits of RATKA. Among patients with mild varus, abnormal MPTA was found in 0% of the RATKA group compared to 8.8% of the CTKA group. In those with severe varus, the abnormal rate was 7.3% in the RATKA group versus 13.6% in CTKA. Notably, FDLA abnormalities were only observed in the severe varus CTKA group (7.6%). These results suggest that RATKA, through preoperative planning and intraoperative precision, facilitates more accurate bone resections and implant positioning. With respect to clinical outcomes, no significant differences were observed in functional scores between groups in mild varus. In contrast, patients with severe varus who underwent RATKA exhibited significantly better postoperative HSS, WOMAC, VAS and ROM scores than those treated with CTKA, further supporting its advantage in managing severe varus patients.

Numerous prior studies have examined the role of RATKA in TKA, with some, including those by Zhang et al. reported superior postoperative outcomes compared to CTKA [36], while others found no meaningful difference [13, 35]. The novelty of our study lies in grouping patients by preoperative HKA angle deviation and demonstrating that the clinical and radiographic benefits of RATKA are particularly pronounced in patients with severe varus, whereas its advantages in mild varus cases are limited. These findings contribute new evidence for refining the clinical indications of RATKA.

Intraoperative analysis of soft tissue releases, including the quadriceps tendon, medial and lateral compartments, and the patella, showed that RATKA, particularly in patients with severe varus, required less soft tissue release compared to CTKA. Appropriate soft tissue balancing is vital for joint mobility, and postoperative stability [28, 29, 33]. RATKA enables optimization of soft tissue tension via 3D preoperative planning and real‐time intraoperative feedback on ligament tension, reducing unnecessary release, minimizing soft tissue trauma, and enhancing postoperative recovery [16, 26]. Proper soft tissue release is of great importance for reduce postoperative pain and promoting good joint range of motion.

Nonetheless, several limitations should be noted in this research. First, the relatively small sample size may limit statistical power and generalizability. Second, the short follow‐up duration precludes assessment of mid‐ to long‐term outcomes. Lastly, surgeon experience and subjective judgement, particularly in evaluating the extent of soft tissue release, may introduce bias. Therefore, future multicenter studies with larger sample sizes and extended follow‐up are warranted to validate these findings.

## CONCLUSION

Compared to CTKA, RATKA confers limited advantage in patients with mild preoperative varus. However, in patients with severe varus, RATKA facilitates more accurate restoration of lower limb alignment, optimized implant positioning, and improved soft‐tissue balance. Based on these findings, the use of RATKA is recommended in patients with severe preoperative HKA angle deviation.

## AUTHOR CONTRIBUTIONS

All authors contributed to the study conception and design. Material preparation, data collection and analysis were performed by Shuaijing Feng, Pengfei Xing and Junsong Qu. The first draft of the manuscript was written by Shuaijing Feng and all authors commented on previous versions of the manuscript. All authors read and approved the final manuscript.

## CONFLICT OF INTEREST STATEMENT

The authors declare no conflicts of interest.

## ETHICS STATEMENT

The study protocol was approved by the Ethics Committee of the First Hospital of China Medical University (Approval No. [2024]1075), and the patients were exempt from informed consent.

## Data Availability

Due to privacy and confidentiality agreements, the raw dataset is not publicly available. Access to anonymized data were granted upon reasonable request to the corresponding author.
